# Sweet-type star fruit supplementation controls oxidative stress status and enhances the community walking capacity among elderly Thai

**DOI:** 10.1186/s12906-023-04291-3

**Published:** 2023-12-11

**Authors:** Jynwara Kaju, Jirakrit Leelarungrayub, Surapol Natakankitkul, James J Laskin

**Affiliations:** 1 Biomedical Sciences Program, Faculty of Associated Medical Sciences, Chiang Mai University, Chiang Mai, 50200 Thailand; 2 Department of Physical Therapy, Faculty of Associated Medical Sciences, Chiang Mai University, Chiang Mai, 50200 Thailand; 3 Pharmaceutical Sciences, Faculty of Pharmacy, Chiang Mai University, Chiang Mai, 50200 Thailand; 4 Department of Occupational Science and Occupational Therapy, Faculty of Medicine, University of British Columbia, Vancouver, British Columbia V6T 1Z4 Canada

**Keywords:** Elderly, Home walking exercise, Oxidative stress, Sweet-type star fruit product

## Abstract

**Background:**

Sweet-type Star fruit (SF) (*Averrhoa carambola* L.) is seasonal and more available for purchase in many markets in Thailand, when compared to the sour-type. But, its antioxidant activity results and potentially more modified supplement for elderly health during regular exercise in the community are unclear.

**Objective:**

This study aimed to evaluate the antioxidant activity and physical capacity from supplementation of sweet-type SF among elderly people performing home walking exercise.

**Methods:**

Mixing SF juice with honey industrially prepared the SF product. Its effects on oxidative stress status and physical capacity were studied in four groups; a supplement with walking exercise (n = 11, 67.00 ± 4.17 years), control (n = 12, aged 67.50 ± 5.58 years), supplementation (n = 11, aged 69.63 ± 7.14 years), and walking exercise (n = 12, aged 67.91 ± 4.33 years). Twenty grams or two teaspoons of supplement in warm water (150 mL) was the guideline for consumption twice daily for 4 weeks. In contrast, the walking exercise was prescribed with moderate intensity for 30 min, 3 days per week. Before and after the 4-week period, the oxidative stress status; glutathione (GSH), ascorbic acid (Vit C), total antioxidant capacity (TAC), and malondialdehyde (MDA), and 6-minute walking distance (6MWD) were evaluated.

**Results:**

Results after the 4-week period, showed that Vit C and TAC increased and the MDA decreased significantly in the supplementation group, except the GSH and 6MWD results. The GSH and Vit C slightly decreased in the walking exercise group, whereas, its TAC, MDA and 6MWD increased significantly. Finally, The GSH and Vit C did not decrease and MDA slightly decreased in the combined group, but, their TAC and 6MWD increased significantly.

**Conclusion:**

Supplementation of the SF product during walking exercise possibly controls oxidative stress status and may enhance walking capacity.

## Background

Nowadays, it is believed that the world population has become an aging society, with many countries having a higher proportion of elderly people. It is thought that 703 million people aged 65 years or over populate the world [1, 2], In 2019, 8,638,000 such people were reported to live in Thailand; a percentage of 12.4%. It has been predicted that the number will increase to over 15.9 million by 2035 [3]. Aging can be described as a degenerative process related to high oxidative stress status [4]. Thus, evaluation of oxidative stress status can be performed from lipid peroxide or malonadiladehyde (MDA), protein carbonyl (PC), and DNA damage, as well as the total antioxidant level [5–7]. Especially, low antioxidant compounds such as glutathione (GSH), alpha-tocopherol (Vit E), L-ascorbic acid and high oxidation on protein, lipid, and nucleic acid in the biological compartment as same as lipoprotein metabolism abnormality have been proposed [8, 9]. Therefore, previous evidence has demonstrated that high oxidative stress induces various chronic diseases such as hypertension, alzheimer, stroke, cardiac, and chronic obstructive pulmonary disease (COPD), etc. [10].

Regular exercise with moderate intensity has been suggested in elderly people. Even though various types of exercise, such as strengthening, endurance and flexibility are can be performed for the elderly [11], cycling, walking and other exercise at home have been recommended [12]. Some reports indicated that community-home exercise either indoor or outdoor walking is a simple and easy program for improving quality of life in either elderly participants or patients with arterial disease [13–15]. However, the intensity of walking should be not too heavy, as that may increase oxidative stress. Thus, a therapeutic approach with antioxidant food or fruit containing multivitamins such as vitamin A, β-carotene, and vitamin C and E [16, 17] has been preferred during exercise [4]. Furthermore, polyphenols have beneficial effect on reducing heart disease, neurodegenerative conditions, cancer and osteoporosis [18]. Therefore, antioxidant nutrient is a challenging choice during home exercise for elderly people, but less evidences have been supported.

Fruits which serve as health promotion and disease prevention, have rich resources of several antioxidant compounds, such as vitamins C, A, and E, and polyphenols [19]. Thailand is located in Asia, with a tropical climate involving heat, humidity and torrential rains [20]. In general, documented data on fruits and vegetables universally promote human health [21]. Some of the tropical fruit species utilized can be grown for commercial marketing or in gardens at home for local markets. Star fruit (SF) (*Averrhoa carambola* L.) is one of many local fruits in Thailand that have specific characteristics. SF has a glassy green skin with sour taste (sour-type), or slightly yellow skin with sweet taste (sweet-type) [22]. Reviewed data showed its various benefits on human health as an appetite stimulant, anti-pyretic, a laxative and diuretic, as well as having digestive effects [23]. Previous reports indicated its active compounds such as ascorbic acid (Vit C), epicatechin, gallic acid (GAE) [24], and flavonoids [25, 26]. Interesting results in previous studies showed that consumption of sour-type SF juice twice daily for 4 weeks could reduce pro-inflammatory cytokines, tumor-necrosis factor-alpha (TNF-α), and interleukin-23 (IL-23), as well as decrease lipid peroxidation and protein oxidation [27]. It also increased the level of high-density lipoprotein (HDL) and reduced low-density lipoprotein (LDL) in elderly subjects [28]. In 2020, this study showed the antioxidant activity of sweet-type SF [26], as well as its beneficial effect on people with COPD after modifying a prototype product composed of sweet-type SF and honey [29]. Unfortunately, the beneficial evidence amng elderly people was not confirmed, especially during their home walking exercise.

## Materials and methods

An experimental protocol study in community dwellings of elderly people was approved by the Ethic Human Committee at the Faculty of Associated Medical Sciences, Chiang Mai University, Thailand (AMSEC-62FB-001). It was conducted in accordance with the Declaration of Helsinki (2001). The protocol in this study had an experimental design on evaluating antioxidant activity of sweet-type SF prototype product as same as in a previous preparation in COPD subjects [29]. The product was prepared by mixing SF juice with pure honey from the flowers of *Chromolaena odorata* (L.) at a ratio of 20:80 (*w:w*), before evaporated until the moisture less than 10%. It was contained in a clean sealed plastic jar (250 g) at the Bee Healthy Company Ltd., Saraphi district, Chiang Mai province, Thailand. The safety of contaminants (chemicals and microbials), main nutrients and Vit C were evaluated preliminarily in the same way as that for contamination with chemicals such as benzoate, sorbic acid, salicylic acid, saccharin and synthetic color, and disease-induced microbials; for instance, most probable number (MPN) Coliforms, *Escherichia coli*, Yeast, Fungi, *Salmonella* sp. *Clostridium perfringens*, *Staphylococcus aureus* and *Bacillus cereus*, which were investigated in the laboratory under the Public Health Guideline (Issue: 356) (2013) of the Department of Medical Sciences, Thailand, before approval for a registration number from the Food and Drug Administration (FDA) in Thailand (Food serial number of 50-1-02237-2-0122).

### Recruitment of participants

#### Subject recruitment

The sample size estimate was performed by G*Power (3.1.9.2) with the effect size of d = 1.34, alpha error prob = 0.05 and power (1-β err prob) = 0.80. According to a preliminary study, 16 healthy subjects showed changes in total antioxidant capacity (TAC) (1.15 ± 0.25 mmol Trolox/L of 8 controls and 1.56 ± 0.35 mmol Trolox/L of 8 consuming subjects) after consuming the pilot honey with the SF concentration in this study. Calculation and analysis by the TAC result with t-test (Means: difference between two independent means) was approved at 10 persons per group. Prevention of loss at 20% also was calculated and it revealed that 2 persons per group should be added. Therefore, 48 participants were required for this study: 4 groups with 12 participants in each group.

The inclusion criteria for elderly people were aged between 60 and 80 years old, living in their community independently and without assistance while performing activities of daily living (ADL), body mass index (BMI) between 18.5 and 23.0 kg/m^2^, with or without stable and controllable underlying disease with medication, and without diagnosed kidney, thyroid or heart disease. The exclusion criteria were severe fever, severe hypertension and urgent severe and uncontrollable underlying disease, consumption of vitamins or other supplementations, and inability to understand instructions. Finally, all of the participants signed a written consent form before the program started. In order to evaluate effectiveness of the supplementary product during the walking exercise, the four groups were formed by the block randomization method; (1) control, (2) supplement product, (3) walking exercise, and (4) combined product and walking exercise by simply picking different number-coded balls from a box (1 = control, 2 = supplement product, 3 = walking exercise, and 4 = combined supplement and walking exercise group).

1. Control group: The participants were asked to maintain their activities and food intake as usual for 4 weeks.
2. Supplement product group: The participants were asked to drink honey with SF concentrated product twice a day in the morning and evening after meals. One teaspoon (10 g) of the product was dissolved in 150 mL of warm water that had an equal yield of L-ascorbic acid at 3.0 mg per drink. Therefore, consumption of 20 g of honey with the SF product was performed every day for 4 weeks.
3. Walking exercise group: The participants were asked to walk around their house with moderate intensity for 30 min, 3 days per week for 4 weeks, as in a previous study. [28] Moderate intensity was determined with a somewhat severe feeling (4 from maximal 10 scalings) from the Borg dyspnea scale [30].
4. Combined supplement and walking exercise group: The participants were asked to follow both the supplement product and walking exercise group. They consumed about 20 g of honey with the SF concentrated product every day for 4 weeks as well as walking at moderate intensity for 30 min, 3 days per week for 4 weeks.

During the 4-week period, all of the participants were required to record their performance in product consumption and/or walking daily in a self-report form. All of the self-report forms and empty product bottles were retrieved at the end of the 4-week experiment. Any participants showing signs or symptoms of side effects from product consumption, such as allergy and severe illness, and those who could not follow instructions, lost consumption of the product or performed less than 20% or 6 days of exercise throughout the 4 weeks, or wanted to stop participating, were excluded from this study. In addition, all of the participants were called by phone each day to check their product consumption, and their exercise was rechecked by their self-report. Village health volunteers were required to visit some of the participants in order to keep in contact. All of the participants were required to maintain their dietary, regular physical activities, including non-permitted to extra-fruits or antioxidant supplements before and during the study period.

### Parameter assay protocols

The day before the 4-week perid and the day after its completion, all of the participants were required to visit the research appointment room, and any food intake after midnight the day before that was not permitted. All of the participants rested on a comfortable chair for 5 min in the temperature room. Ten mL of blood was taken from the anterior cubital vein by a medical technologist (MT) and separated into a 3 mL sodium fluoride tube for fasting blood sugar (FBS) and triglyceride tests, a 2 mL ethylene diamine tetraacetic acid (EDTA) tube for complete blood cell (CBC) tests, and a 3 mL clotted blood tube for renal and liver function tests. Finally, 2 ml of blood was kept in the EDTA tube for oxidative stress evaluation (GSH, Vit C, TAC, and MDA). From the 2 mL of whole blood, 400 µL was separated in order to evaluate the GSH, with residual blood being centrifuged at 3,000 rpm for 10 min to separate the plasma, which was taken to the laboratory to determine the TAC, MDA and Vit C.

The GSH was evaluated with a modified method of Ellman’s reagent [27]. A 400 µL sample of whole blood was prepared by adding 3 mL of dH_2_O and 1.6 mL of precipitant solution. After waiting 5 min before centrifuging at 10,000 rpm for 2 min, only the supernatant was used. Then, 200 µL of clear supernatant was mixed with 500 µL of phosphate solution (PBS) and left for 5 min. When the sample turned yellow, it was measured by spectrophotometry at 412 nm. The GSH unit was expressed as milligrams per gram of hemoglobin and individually compared to standard GSH (mg/g Hb).

Vit C was evaluated under high performance liquid chromatography (HPLC) [31]. In brief, 100 µL of plasma was precipitated with 400 µL of 60% ethanol containing EDTA (1.0 mmol/L). After 200 µL of supernatant was evaporated by high-speed centrifugation, 100 µL of ethanol containing 100 µg of standard Vit C was resolved, before identifying Vit C under the HPLC system (Eclipase Plus C18 Column, 5 μm, 4.6 × 250 mm Agilent, USA; 0.1% formic acid, Sigma-Aldrich, Germany; 0.8 mL/min of flow rate, and 244 nm absorbance). The Vit C in plasma was calculated by comparing with the standard curve peak of Vit C (Fisher Scientific, UK).

The TAC was evaluated by following the modified protocol in a previous study [28] under the original 2, 2-azino-bis-3-ethylbenzothiazoline-6-sulfonic acid (ABTS) decolorization method [32]. The TAC of plasma on scavenging ABTS^•+^ was produced by reacting ABTS (CALBIOCHEM, Darmstadt, Germany) with potassium persulfate (Merck KGaA, Darmstadt, Germany) in deionized water. Initial absorbance at 990 µL of ABTS^•+^, and at 734 nm and 0.7 ± 0.02, was set before adding 10 µL of plasma. The percentage of maximal reduction of absorbance was calculated by spectrophotometry. Then, the plasma TAC was represented as millimoles of standard Trolox per liter of plasma (mmol Trolox/L).

The MDA from lipid oxidation was evaluated by following a previous method [33], with the protocol of spectrophotometry under the thiobarbituric acid reactive substances (TBARs) test. A 200 µl of plasma was mixed with 750 µl of ortho-phosphoric acid (2.5%, v/v) and 200 µL of thiobarbituric acid (0.2 mol/L) solution. After 30 min of heating at 90 °C, short high-speed centrifugation was carried out at 10,000 rpm in room temperature in order to remove all pellets. Absorbance of clear yellowed supernatant of TBARs-MDA adduct was read by spectrophotometry, and expressed as equal to the standard tetra-methoxypropane (Sigma-Aldrich Co.).

The 6MWD was evaluated from the standardized guide of the American Thoracic Society protocol [34], which was modified from the 20-meter straight walking test performed in an outside corridor. Any vigorous physical activities and eating 2 h before starting the 6MWD were avoided under the testing recommendation of the American College Society of Medicine [35]. Comfortable clothes and shoes could be worn during the test, and vital signs such as heart rate, blood pressure, respiratory rate and oxygen saturation were evaluated for safety reasons before and after the test.

### Health and adverse effects evaluation

CBC, renal function (blood urea nitrogen; BUN and creatinine), liver function (aspartate aminotransferase; AST; alanine aminotransferase; ALT), and FBS were evaluated for adverse effects from product consumption, and standardized and performed by a fully automated Olympus AU400 Analyzer (Olympus Diagnostics GmbH, Umkirch, Germany) at the Bangkok R.I.A. Laboratory, Chiang Mai, Thailand. In addition, all symptomatic adverse effects such as vomiting, confusion, vertigo, numbness or hiccups, with five categories of symptoms (none, mild, modereate, severe or very severe) were ascertained at an interview before and after completion of the 4-week protocol [29].

### Statistical analysis

All of the data were analyzed statistically for normal distribution using the One-sample Shapiro-Wilk test before presenting as mean, with standard deviation, including and minimal and maximal value. All parameters at baseline or during the 4-week period to completion were analyzed statistically between the 4 groups by the non-parametric Kruskal-Wallis Test. Whereas, the statistical difference between the baseline and after completing the 4-week period was evaluated within each group using the non-parametric Least Significant Difference (LSD) test. All statistical analyses were performed using the SPSS version 17.0 (SPSS Inc, Chicago, IL, USA) for Windows. All of the tests were used with significance at p < 0.05.

## Results

### Participant data

From the 48 elderly participants recruited in the program, one of them in the combined supplement product and walking exercise group withdrew because of the inconvenient time of the study. Therefore, 12, 12, 12 and 11 participants in the control, supplement product, walking exercise and combined supplement product and walking exercise group, respectively, completed protocol. When the characteristic data of all the groups were distributed, and analyzed using the Shapiro-Wilk Test, normal distribution was presented in all characteristics of the four groups (control, supplement, walk, and combined supplement and walk); age (0.27, 0.06, 0.63, and 0.95, respectively), weight (0.56, 0.09, 0.45, and 0.86, respectively), height (0.72, 0.36, 0.277, and 0.09, respectively) and BMI (0.34, 0.56, 0.67, and 0.21, respectively). Thus, the mean and standard deviation of each datum are shown in Table 1. In addition, all characteristics between the four groups were not statistically different (p > 0.05) from those in the K-independent paired analysis (Kruskal-Wallis H test).

**Table 1:** Characteristics of the elderly participants in each group

Parameters	Control (n =12)	Supplement (n =11)	Walk Exr. (n=12)	Supplement + Walk Exr. (n=11)	P
n (male: female)	12 (6:6)	11 (7:4)	12 (6:6)	11 (7:4)	
Age (years)	67.50 ± 5.58 (61-77)	69.63 ± 7.14 (61-80)	67.91 ± 4.33 (61-75)	67.00 ± 4.17 (61-72)	0.85
Weight (kg)	57.50 ± 3.73 (52-68)	55.40 ± 3.98 (59-70)	58.21 ± 3.41 (57-75)	55.25 ± 3.26 (55-64)	0.69
Height (m)	1.65 ± 0.05 (1.54-1.71)	1.62 ± 0.05 (1.55-1.70)	1.62 ± 0.07 (1.48-1.70)	1.64 ± 0.05 (1.55-1.70)	0.52
BMI (kg.m^-2^)	22.46 ± 1.35 (19.5-22.9)	21.53 ± 1.02 (18.5- 22.6)	22.14 ± 0.94 (18.6-22.8)	21.05 ± 1.25 (19.5-22.6)	0.73

*Note*: Data present a mean and standard deviation (SD). BMI = body mass index. The p value was analyzed statistically with the Kruskal-Wallis H Test

### Oxidative stress status

The group with the main outcome of oxidative stress status in the 4 weeks was the combination of supplement product and walking exercise group (S + W), when compared to the control (C), supplement product (S) and walking exercise (W) groups. All of the parameters in each group at baseline or after completing the 4-week period are presented in Table 2. Normality was rechecked by the Spiro-Wilk test, which showed normal distribution in the baseline period; GSH (p = 0.13, 0.78, 0.22, and 0.58), Vit C (p = 0.85, 0.28, 0.84, and 0.52), TAC (p = 0.75,0.28, 0.62, and 0.57), and MDA (p = 0.44, 0.74, 0.22, and 0.93), and also in the post 4-week period; GSH (p = 0.87, 0.14, 0.69, and 0.59), Vit C (p = 0.66, 0.53, 0.87, and 0.64), TAC (p = 0.06, 0.79, 0.08, and 0.99), and MDA (p = 0.56, 0.45, 0.06, and 0.19). Therefore, the mean with standard deviation (minimal and maximal values) was represented.

**Table 2:** Oxidative Stress Status in the four groups

Parameters	Control (n =12)		Supplement (n= 11)		Walk Exr. (n = 12)		Supplement + Walk Exr. (n =11)		p^*^	p^#^
	Before	After	Before	After	Before	After	Before	After		
GSH (mg/g Hb)	12.38 ±1.24 (10.2-14.2)	12.24 ± 0.22 (10.1-13.8)	12.48 ± 1.26 (11.0-15.1)	12.35 ± 1.34 (10.3-14.5)	11.9 ± 1.45 (10.1-14.2)	10.49 ± 1.19 (8.5-13.2)	12.05 ± 1.65 (10.2-15.1)	11.71 ± 1.54 (9.2-14.6)	0.67	0.01
Ascorbic acid (µg/dL)	66.90 ± 7.63 (56.3-80.6)	66.69 ±8.36 (55.2-79.5)	66.03 ± 8.42 (54.3-81.3)	69.90 ± 9.43 (56.2-86.5)	66.70 ± 11.54 (45.3-82.16)	60.42 ± 9.49 (42.3-75.6)	66.08 ± 8.77 (55.6-78.44)	68.25 ± 7.45 (57.2-80.5)	0.99	0.12
TAC (mmol Trolox/L)	1.16 ± 0.29 (0.56-1.0)	1.18 ± 0.24 (0.8-1.6)	1.12 ± 0.32 (0.67-1.80)	2.13 ± 0.82 (1.10-1.96)	1.04 ± 0.22 (0.70-1.40)	1.26 ± 0.28 (0.76-1.80)	1.01 ± 0.23 (0.60-1.40)	1.13 ± 0.78 (0.80-1.80)	0.76	0.00
MDA (µmol/L)	3.05 ± 0.6 (2.1-4.1)	2.96 ± 0.59 (2.1-3.9)	2.9 ± 0.49 (2.1-3.5)	2.16 ± 0.51 (1.6-3.2)	3.05 ± 0.59 (2.1-4.1)	3.41 ± 0.67 (2.4-4.8)	3.02 ± 0.48 (2.5-4.2)	2.88 ± 0.31 (2.5-3.5)	0.93	0.00
6MWD (m)	320.2 ±11.2 (258-305)	321.4 ± 10.4 (262-313)	311.45 ± 5.74 (260-325)	320.5 ± 13.11 (242-336)	309.5 ± 10.38 (230-345)	354.2 ± 9.41 (282-430)	320.3 ±11.21 (255-315)	376.7 ±13.51 (288-510)	0.72	0.01

*Note*: GSH = glutathione, TAC = total antioxidant capacity, MDA = malondialdehyde, 6MWD = 6-minute walking distrance, *p value was calculated statistically between the four groups at baseline or before, and #p value was calculated statistically between the four groups after the 4-week period with the K-independent Kruskal-Wallkis H test

Statistical difference of each parameter between the groups was performed by the non-parametric Kruskal-Wallis H test. All parameters showed no statistical difference between the 4 groups at the baseline period; GSH (p = 0.67), Vit C (p = 0.99), TAC (p = 0.76) and MDA (p = 0.93), respectively. Whereas, a statistical difference was shown between the 4 groups at the end of the 4-week period; GSH (p = 0.01), Vit C (p = 0.02), TAC (p = 0.00) and MDA (p = 0.00), respectively.

**Fig. 1:**
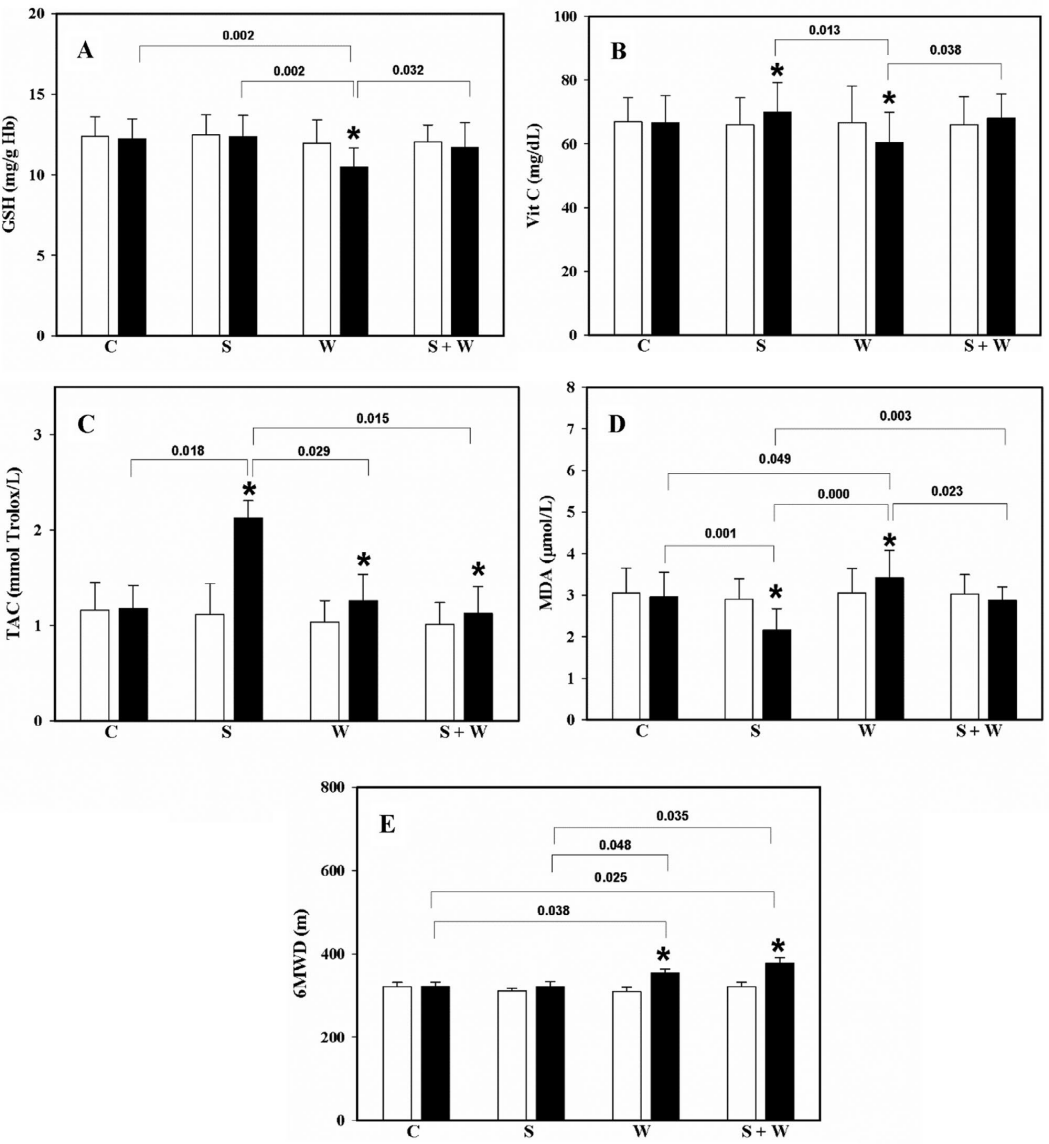
Oxidative stress status; **A**: glutathione (GSH), **B**: L-ascorbic acid (Vit C), **C**: total antioxidant capacity (TAC), **D**: malondialdehyde (MDA), and **E**: 6-minute walking distance (6MWD). Data represent mean and standard deviation at baseline (white bar) and after completion of the 4-week period (black bar) between the control (C), supplement product (S), walking exercise (W) and supplement product and walking exercise (S + W) groups

After observing the results in each parameter, the GSH level in the walking exercise group was reduced when compared to the baseline, and also lower than the other groups (p < 0.05). Whereas, the combined supplement product and walking exercise group showed a slightly decreased level, with no statistical difference (Fig. 1A).

The Vit C level increased significantly in the supplement product group when compared to the baseline (p = 0.008), but it decreased in only the walking exercise group (p = 0.002). In addition, the Vit C level in the walking exercise group was significantly lower than that in the combined supplement product and walking exercise group (p = 0.013 & 0.038), but no different from the control group. However, the supplement product and walking exercise group showed a slightly increased Vit C level, without a statistical difference (p = 0.06) (Fig. 1B).

The level of TAC in three of the groups significantly increased when compared to the baseline after completing the 4-week protocol; supplement product (p = 0.004), walking exercise (p = 0.013), and supplement product and walking exercise (p = 0.032). The supplement product group presented the highest TAC level when compared to the control (p = 0.018), walking exercise (p = 0.029) and supplement and walking exercise groups (p = 0.015). Finally, walking with or without supplementation did not present a statistical difference (p = 0.74) (Fig. 1C).

Result of the MDA level showed that only the supplement product group reduced lipid peroxide in plasma significantly when compared to its baseline (p = 0.003), and also compared to the other groups; control (p = 0.001), walking exercise (p = 0.000), and supplement product and walking exercise (p = 0.003). The walking exercise group showed a significantly higher MDA level (p = 0.022) during the 4 weeks when compared to its baseline and the other groups; control (p = 0.049), supplement product (0.000) and supplement product and walking exercise (0.023). Although, the supplement product and walking exercise group showed a slightly decreased MDA level (p = 0.229) and significantly different one from the walking exercise and supplement product group, there was no significant difference when compared to the control (p = 0.71) (Fig. 1D).

Finally, the result of physical function from the 6MWD showed no statistical difference in the control or supplement product group. The walking group statistically increased their distance with (p = 0.03) or without (p = 0.02) supplementation, when compared to the baseline period. The walking exercise group also presented a longer distance when compared to the other groups; control (p = 0.038), supplement product (p = 0.048), and supplement product and walking exercise (p = 0.035). However, the walking exercise group showed no statistical difference (p = 0.56) with or without supplementation (Fig. 1E).

### Health and adverse effects status

Results of CBC, renal function, liver function, and FBS tests presented the health status or some adverse effects from product supplementation, which are presented in Table 3. Results of all parameters; CBC, renal and liver functions and FBS showed no statistical difference between baseline and after the 4-week period (p > 0.05). Although, FBS in the supplement product and supplement product and walking exercise group had no significant increase, it was in the normal reference ranges. Moreover, other adverse or side effects such as vomiting, confusion, vertigo, numbness or hiccups were not reported in the supplement or supplement with walking exercise group over the 4-week study period.

**Table 3:** Health status and adverse effects on complete blood count (CBC), renal and liver function, and fasting blood suger (FBS) tests

Parameters	Control (n=12)		Supplement (n=11)		Walk Exr. (n=12)		Supplement + walk Exr. (n =11)		p*	p^#^
	Before	After	Before	After	Before	After	Before	After		
CBC										
WBC (10^3^/µL)	5.5 ± 1.2 (5.1-9.5)	5.8 ± 1.0 (4.6-9.5)	5.9 ± 1.5 (5.6-9.2)	6.1 ± 1.1 (5.4-9.9)	5.1 ± 1.1 (4.5-10.2)	5.95 ± 1.3 (5.5-9.7)	6.1 ± 1.5 (5.5-10.6)	6.6 ± 1.2 (4.7-9.0)	0.45	0.38
RBC (10^6^/µL)	4.5 ± 0.5 (3.9-5.1)	4.9 ± 0.9 (4.0-5.2)	4.3 ± 0.2 (3.8-5.6)	4.1 ± 0.2 (3.3-5.2)	4.8 ± 0.2 (4.2-5.0)	4.6 ± 0.3 (4.1-5.0)	4.1 ± 0.3 (3.4-4.8)	4.9 ± 0.7 (3.2-5.3)	0.61	0.51
Hb (g/dL)	13.1 ± 1.4 (11.2-15.5)	12.4 ± 1.5 (10.1-15.2)	15.2 ± 1.8 (10.6-15.8)	14.7 ± 1.4 (11.6-15.1)	12.7 ± 1.3 (10.4-14.0)	13.0 ± 1.2 (10.2-15.0)	13.5 ± 1.6 (12.1-15.2)	14.2 ± 1.3 (11.3-15.3)	0.35	0.52
Hct (%)	41.2 ± 1.5 (35.2-44.5)	44.2 ± 1.3 (36.1-44.2)	39.1 ± 1.2 (36.2-48.0)	41.1 ± 1.1 (35.9-43.2)	39.9 ± 1.2 (36.1-45.2)	40.2 ± 1.5 (37.1-49.5)	39.6 ± 1.2 (36.0-43.2)	44.1 ± 1.0 (35.3-43.1)	0.22	0.34
PLT (10^3^/µL)	235 ± 32.5 (145-345)	241 ± 28.5 (140-325)	256 ± 29.4 (141-410)	230 ± 30.1 (145-399)	225 ±33.2 (139-412)	234 ± 32.5 (141-392)	230 ± 19.3 (144-385)	239 ± 28.5 (140-401)	0.78	0.65
**Renal function**										
BUN (mg/dL)	13.4 ± 0.9 (9.9-18.0)	12.1 ± 0.7 (9.8-16.5)	12.2 ± 0.4 (7.5-16.3)	13.8 ± 0.2 (7.1-6.1)	12.7 ± 1.1 (6.8-16.5)	12.1 ± 0.9 (7.3-16.2)	14.1 ± 0.3 (9.9-18.8)	14.4 ± 1.1 (9.5-16.4)	0.65	0.54
Creatinine (mg/dL)	0.9 ± 0.0 (0.6-1.1)	0.8 ± 0.1 (0.6-1.2)	0.7 ± 0.0 (0.5-1.1)	0.5 ± 0.1 (0.7-1.1)	0.8 ± 0.2 (0.6-1.3)	0.8 ± 0.1 (0.7-1.4)	0.9 ± 0.0 (0.6-1.1)	0.8 ± 0.2 (0.7-1.2)	0.80	0.73
**Liver function**										
AST (U/L)	24.7 ± 2.9 (13-33)	22.7 ± 2.3 (11-31)	26.2 ± 2.2 (15-33)	27.1 ± 2.3 (14-35)	22.8 ± 2.1 (15-29)	21.2 ± 1.9 (16-28)	24.5 ± 2.0 (11-32)	26.2 ± 3.1 (12-30)	0.64	0.55
ALT (U/L)	22.4 ±1.5 (12-31)	20.9 ± 2.1 (11-30)	23.1 ± 2.0 (14-28)	23.8 ± 1.5 (16-31)	20.9 ± 3.4 (10-30)	21.5 ± 2.1 (14-28)	22.3 ± 1.9 (15-27)	25.2 ± 1.5 (10-26)	0.56	0.45
FBS (mg/dL)	91.7 ± 1.2 (78-99)	90.6 ± 2.2 (77-100)	88.2 ± 2.3 (80-98)	92 ± 3.4 (78-99)	96.8 ± 2.3 (77-100)	92.9 ± 1.5 (79-87)	90.1 ± 2.4 (83-100)	94.2 ± 3.2 (70-99)	0.89	0.61

*Note*: WBC = white blood count, RBC = red blood count, Hb = hemoblogin, Hct = hematocrite, PLT = platelet, BUN = blood urea nitrogen, AST = aspartate transferease, ALT = alanine transferase, FBS = fasting blood suger. * p value was calculated statistically between the four groups at baseline or before, and # p value was calculated statistically between the four groups after completion of the 4-week period with the K-independent Kruskal-Wallkis H test

## Discussion

This was a pilot study of a supplementary product prepared by standardized manufacture from seasonal sweet-type SF and honey in a community of elderly people. Two types of SF have been distributed in Thailand; sweet- and sour-type. Most sweet-type are popularly purchased in markets, whereas the sour-type is cultivated in local farms. In 2016, the beneficial effects of consuming sour-type SF juice for 4 weeks were shown in elderly Thai people. Antioxidant and anti-inflammatory activity, and enhanced physical walking capacity with high Vit C and total phenolic compound were reported [27, 28]. Moreover, the 4-week supplement of sour-type SF juice could reduce TNF-∝ and NO in elderly people [27]. Whereas, the sweet-type was studied less in Thailand. Updated evidence on sweet-type SF extract showed activities on radical scavenging as well as suppressed inflammation through inhibiting TNF-∝ in vitro [26]. Thus, the new product of sweet-type SF was very challenging at the industrial level as in previous study [29]. The SF product was prepared similarly under the guideline of the Department of Public Health, Thailand, before being approved for the study of people with COPD in that study and in a community of elderly people in this study. Contamination from yeast, fungi [36], microbial pathogens, especially coliforms, *Escherichia coli*, yeast, fungi, *Salmonella* sp, *Clostridium perfringens*, *Staphylocossus aureus* and *Bacillus cereus*, placed under a compendium of methods for food analysis [37], was approved with a food serial number of 50-1-02237-2-0122. Moreove, possible toxicity from oxalic acid content in the SF product in this study, which may affect neurotoxic or neuphrotoxic conditions [38] was rechecked as in the previous study [29]. Previous evidence reported that a minimal dose of 4–5 g of oxalate caused death in adults [39]. The product for this study was only 0.625 milligrams in 100 mg [29] which is very low content when administered at only 20 g per dose. Therefore, the oxalic acid dose was very safe in this study as in the previous one [29].

Previous evidence of the SF product in this study was found in people diagnosed with stable COPD [29] because of their high oxidative stress and chronic inflammation. [40, 41] However, interesting evidence of its beneficial effects in healthy elderly people or people without disease was still unclear and needed to be confirmed, especially on antioxidant status. Thus, GSH, Vit C, TAC and MDA were evaluated in this study.

The main design of this protocol was to concentrate on the combination of SF product consumption with walking exercise at home because the previous study documented that exercise can improve the physical capacity and quality of health among COPD patients [42] by reducing MDA and improving the total radical-trapping antioxidant parameter (TRAP) after an 8-week conditioning program with a cycle ergometer (three times per week) [43]. However, a cycle ergometer was not available at homes with low-economic status, thus a very simple home-based walking exercise was performed [44] in order to improve quality of life (QOL) and exercise tolerance [45, 46]. A 20-minute period of walking exercise, by following the previous study, was recommended for patients with moderate dyspnea in order to improve their exercise capacity and QOL [46].

However, contrasting reports suggested that the walking exercise can be harmful, especially in people with chronic disease such as COPD. For instance, localized leg quadriceps exercise increased MDA levels and oxidative stress index, and reduced the vitamin E level in COPD patients, when compared to healthy subjects [47, 48]. Although, walking exercise at home potentially benefits physical activity and QOL, any benefits on oxidative stress among sedentary people is controversial. This hypothesis was confirmed by the results in this study, which found the GSH and Vit C levels significantly depressed, whereas the MDA level increased. This means that the walking exercise for 4 weeks involves oxidative stress status in sedentary elderly people (Fig. 1). In addition, supplementation of the SF product showed antioxidant status with higher Vit C and TAC status, and significantly decreased MDA level, with no effect on the GSH level in the blood. These results supported the previous report that proposed “application of antioxidant therapy could improve the exercise time and decrease the MDA level in ten COPD patients” [49]. Furthermore, an updated study in 2019 showed that oral antioxidant supplementation, comprising tocopherol, ascorbate, zinc gluconate, and selenomethionine during 28 days of moderate-intensity exercise training, increased muscle strength, serum protein, and plasma antioxidant compounds in COPD patients, when compared to non-antioxidant supplementation during exercise training [50].

In this study, the product consisted of sweet-type SF juice mixed with honey at a 1:4 ratio by weight, of which the optimized Vit C dose of 20 g per day might not be enough for antioxidant activity. However, a low concentration of Vit C was identified in the product as in the previous result [29]. The honey base in the product indicated health benefits such as debriding wounds, killing bacteria, penetrating biofilm, lowering wound pH, reducing chronic inflammation, and promoting fibroblast infiltration [51], with bioactive compounds of octadecanoic acid [52] and oligosaccharides, amino acid, protein, phenolics and flavonoids [53].

Previous evidence suggested that the honey presented anti-lipid oxidation activity [54] because of its total phenolic compound [55]. In addition, a previous report showed that the efficiency of buckwheat honey containing total phenolic compound increased plasma antioxidant and reduced capacity among healthy subjects [56]. Therefore, the product in this study, which is composed of SF juice and honey, possibly has antioxidant activity. This hypothesis supports the results of Vit C and MDA level in this study, and also those in the previous document [57].

The results of small Vit C yield in the product, and whether it involves or effects physical function is interesting. A previous study suggested that 3 g of Vit C supplement could prevent muscle damage and increase exercise time [58]. Thus, this result cannot be summarized, in that this product supplementation can improve physical function because of very low Vit C in the product, in addition to the pro-oxidant activity at a high concentration from in vitro study in elderly subjects. Although, slightly more distance was achieved in the supplement product and walking exercise group, a non-signficant difference was presented. The small sample size in all of the groups possibly may be the main factor, therefore, a larger sample size must be found in future study. However, the non-significant change in walking distance from 309.5 ± 10.38 m to 354.2 ± 9.41 m in the walking exercise group and from 320.3 ± 11.21 m to 376.7 ± 13.51 m in the supplement product and walking exercise group was recorded. The average difference in distance of 56.4 m in the supplement product and walking exercise group was compared to 44.7 m in the walking exercise group. This indicates minimal clinical improvement in physical function, which refers to within 54–80 m in the previous proposal [59].

Finally, this study confirmed the health status or adverse effects from the SF with honey product. All parameters showed no statistical difference between baseline and after completion of the 4-week period, especially regarding kidney function (normal reference range at 5–20 mg/dL of BUN and 0.6–1.5 mg/dL of creatinine) [60]. In addition, the high glucose level in the SF product with honey also should be realized in cases of diabetes or glucose-induced diabetes. Nutritional facts from the United States Drug Association (USDA) showed 5.3 g of glucose in 132 g of cubed SF [61]. Thus, the FBS level was rechecked, and results showed a slight increase without statistical changes in both groups using the supplement product when the 4-week period was completed. Although the honey was mixed with SF juice, the FBS level was not statistically different. This result is safety supported by a previous study of diabetic patients with no statistical difference from 8 weeks of honey consumption [62].

Moreover, there were no symptoms reported by any of the participants who consumed the product. A possible reason for this could be the low amount of SF in the product, which is supported by a previous report [63]. Adverse effects from consumption of SF such as hiccups, vomiting, insomnia and mental confusion could be presented when pure high-dose SF is taken at 1,500 mL per day. In added data from interviews on other symptoms; vomiting, confusion, vertigo, numbness, or hiccups were not reported within the 4-weeks of study, which possibly confirms no side effects from carmbosin toxicity in the central nervous system [64]. Furthermore, this study could not confirm whether there was biological toxicity, therefore, more study and confirmation are needed, especially on longer consumption time, and larger sample size.

## Conclusion

This study showed that a prototype product of sweet-type SF mixed with honey showed antioxidant activity by increasing the Vit C and TAC, and also decreasing lipid peroxide in the human body. Applying a routine walking exercise could protect Vit C and depress MDA production among sedentary elderly people.

## Limitation and suggestion

Although, this study was performed in elderly people living in a community, and showed positive antioxidant effects, it had a small sample size of 11 to 12 people in each group and the benefits on physical capacity could not be exactly concluded. Thus, a larger sample size must be studied in the future.

## Data Availability

Not applicable.
